# Calpain Inhibition Reduces Amplitude and Accelerates Decay of the Late Sodium Current in Ventricular Myocytes from Dogs with Chronic Heart Failure

**DOI:** 10.1371/journal.pone.0054436

**Published:** 2013-04-15

**Authors:** Albertas Undrovinas, Victor A. Maltsev, Hani N. Sabbah

**Affiliations:** 1 Department of Internal Medicine, Henry Ford Hospital, Detroit, Michigan, United States of America; 2 National Institute on Aging, Intramural Research Program, Baltimore, Maryland, United States of America; Georgia State University, United States of America

## Abstract

Calpain is an intracellular Ca^2+^ -activated protease that is involved in numerous Ca^2+^ dependent regulation of protein function in many cell types. This paper tests a hypothesis that calpains are involved in Ca^2+^ -dependent increase of the late sodium current (I_NaL_) in failing heart. Chronic heart failure (HF) was induced in 2 dogs by multiple coronary artery embolization. Using a conventional patch-clamp technique, the whole-cell I_NaL_ was recorded in enzymatically isolated ventricular cardiomyocytes (VCMs) in which I_NaL_ was activated by the presence of a higher (1μM) intracellular [Ca^2+^] in the patch pipette. Cell suspensions were exposed to a cell- permeant calpain inhibitor MDL-28170 for 1–2 h before I_NaL_ recordings. The numerical excitation-contraction coupling (ECC) model was used to evaluate electrophysiological effects of calpain inhibition in silico. MDL caused acceleration of I_NaL_ decay evaluated by the two-exponential fit (τ_1_ = 42±3.0 ms τ_2_ = 435±27 ms, n = 6, in MDL vs. τ_1_ = 52±2.1 ms τ_2_ = 605±26 control no vehicle, n = 11, and vs. τ_1_ = 52±2.8 ms τ_2_ = 583±37 ms n = 7, control with vehicle, P<0.05 ANOVA). MDL significantly reduced I_NaL_ density recorded at –30 mV (0.488±0.03, n = 12, in control no vehicle, 0.4502±0.0210, n = 9 in vehicle vs. 0.166±0.05pA/pF, n = 5, in MDL). Our measurements of current-voltage relationships demonstrated that the I_NaL_ density was decreased by MDL in a wide range of potentials, including that for the action potential plateau. At the same time the membrane potential dependency of the steady-state activation and inactivation remained unchanged in the MDL-treated VCMs. Our ECC model predicted that calpain inhibition greatly improves myocyte function by reducing the action potential duration and intracellular diastolic Ca^2+^ accumulation in the pulse train.

**Conclusions:**

Calpain inhibition reverses I_NaL_ changes in failing dog ventricular cardiomyocytes in the presence of high intracellular Ca^2+^. Specifically it decreases I_NaL_ density and accelerates I_NaL_ kinetics resulting in improvement of myocyte electrical response and Ca^2+^ handling as predicted by our in silico simulations.

## Introduction

The role of the late sodium current (I_NaL_) in electrophysiological remodeling and arrhythmias in chronic heart failure (HF) has been extensively studied during the last decade. It has been shown that I_NaL_ is augmented and its decay slowed in failing human and dog ventricular cardiomyocytes (VCMs)(see for review [1]). A remarkable contribution of I_NaL_ into HF mechanisms has been demonstrated in experiments where “correction” of I_NaL_ in failing VCMs resulted in: 1) rescue of normal repolarization, 2) decrease beat-to-beat action potential (AP) duration variability, and 3) improvement of Ca^2+^ handling and contractility [1]. Accordingly, I_NaL_ has emerged as a novel target for cardioprotection to treat the failing heart [1], [2] The new approaches may involve: 1) discovery new drugs that directly and specifically target I_NaL_, 2) targeting intracellular signaling pathways (for example Ca^2+^-dependent signaling) that are altered in HF and may have modulatory effect on I_NaL_, 3) modulation of altered Na^+^ channel (NaCh) microenvironment, such as different expression of auxiliary β-subunits and sub-sarcolemmal cytoskeleton that, in turn, may be responsible for the augmented slowed I_NaL_ in HF, 4) combination of two latter mechanisms. The new drug, ranolazine (RAN) that was developed as an antianginal agent, has been demonstrated to specifically inhibit I_NaL_ [3], [4]. RAN reduced arrhythmias in the immediately post-MI patients in the recent MERILIN-TIMI trial [5] confirming the clinical relevance of I_NaL_. Ca^2+^, calmodulin and CaMKII and this Ca^2+^ signaling pathway can significantly amplify I_NaL_ in HF affecting both contractile and electrical performance [6], [7]. As to NaCh microenvironment, it has been shown that alterations in membrane phospholipids composition and/or in sub-sarcolemmal cytoskeleton, which consists of ankyrin, actin, spectrin (fodrin), can affect NaCh gating in heart in the way that the late openings may occur [1], [8], [9]. Recently we have shown that silencing SCN1B but not SCN2B, the genes that are responsible for expression of the β_1_ and β_2_ NaCh subunits, could be a plausible mechanism to modulate I_NaL_ in HF with the aim to improve both contractility and rhythm [10].

Calpain is an intracellular Ca^2+^ -activated protease and an important mediator of the actions of the intracellular Ca^2+^ in heart. Cleavage by calpain is critical in a variety of calcium-regulated cellular processes such as muscle contraction, neuronal excitability, secretion, signal transduction, cell proliferation, differentiation, cell cycle progression, and apoptosis [11], [12]. Deregulation of calpain caused by impaired Ca^2+^ homeostasis during cardiac pathologies such as atrial fibrillation, heart failure, hypertrophy, or ischemia reperfusion, is critically involved in the myocardial damage. One of the intracellular targets of calpain is fodrin, a dynamic structure that is altered under a variety of pathological conditions featuring poor Ca^2+^ handling (e.g. ischemia or heart failure [13], [14], [15], [16]). In the present study we tested the hypothesis that the membrane-permeant calpain inhibitor MDL-28170 (MDL) can prevent, in part, Ca^2+^-related I_NaL_ modulation in VCMs from dogs with chronic HF. We found that MDL reduces density of whole-cell I_NaL_ and makes I_NaL_ decay faster in the failing VCMs. Using the excitation – contraction coupling (ECC) numerical model [17] we also assessed physiological significance of the MDL effects. We show that these MDL-induced I_NaL_ alterations: 1) reduce AP duration, and 2) prevent diastolic intracellular Ca^2+^ accumulation during the excitation pulse train in silico.

## Materials and Methods

### 2.1. HF model and cardiomyocyte isolation

The study conforms to the Guidelines for Care and Use of Laboratory Animals published by the US National Institutes of Health and was approved by the Animal Care and Use Committee (IACUC protocols 0816 and 0777) of the Henry Ford Health System. Chronic heart failure that is similar by vast array of functional and pathophysiological parameters [18] to that in humans was produced in 2 dogs by multiple sequential coronary artery microsphere embolizations as previously described [19]. At the time of harvesting the heart (∼3 months after last embolization), left ventricular (LV) ejection fraction was approximately ∼25%. Ventricular cardiomyocytes (VCMs) were enzymatically isolated from the apical LV mid-myocardial slices as previously reported [20]. The yield of viable rod-shaped, Ca^2+^-tolerant VCMs varied from 40 to 70%.

### 2.2. Patch clamp technique and data analysis

I_NaL_ was measured using a whole-cell patch-clamp technique [20]. I_NaL_ was assessed by 2 s-long membrane depolarizations to various potentials from a holding potential of −130 mV applied with a stimulation frequency of 0.2 Hz. The bath solution contained (in mM):140 NaCl, 5.0 CsCl, 1.8 CaCl_2_, 2.0 MgCl2, 5 glucose, 0.002 nifedipine, and 5 HEPES-CsOH buffer (Ph 7.4). The pipette solution contained (in mM): 5 NaCl, 133 CsCl, 0.9 CaCl_2_ (free [Ca^2+^] = 1 μM) MgATP, 20 Tetraethylammonium chloride, 1.0 EGTA, and 5.0 HEPES-CsOH buffer. The free [Ca^2+^] of 1 μM was set in the pipette (and hence inside the cell) to exaggerate abnormal effect of Ca^2+^ on I_NaL_ in HF [21]. Experiments were performed at room temperature (22–24°C). A stock solution of the cell-permeant MDL 28170 (MDL) was prepared in DMSO. MDL was then diluted in the bath solution to a final concentration of 0.2 μM and 2.6 mM DMSO [21], [22]. Cells suspensions were exposed to MDL from 1–2 hours prior to patch-clamp experiments, and MDL was also added to the pipette solution [23], [24]. All measurements were made in the presence of MDL in the bath solution and 8–25 min after the membrane rupture to complete cell dialysis with intracellular recording solutions [25], [26].

The time course of I_NaL_ decay has been approximated by a double exponential fit to I_NaL_ starting at 40 ms after the onset of depolarization to –30 mV as previously suggested [27]: 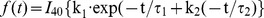
(1)where *τ_1_* and *τ_2_* are the time constants, *I_40_* is I_NaL_ instant value 40 ms after membrane depolarization, *k_1_* and *k_2_* are the contributions of each exponents (*k_1_ + k_2_*  = 1), respectively. 5-15 experimental traces were averaged to improve the quality of analysis.

**Figure 1:**
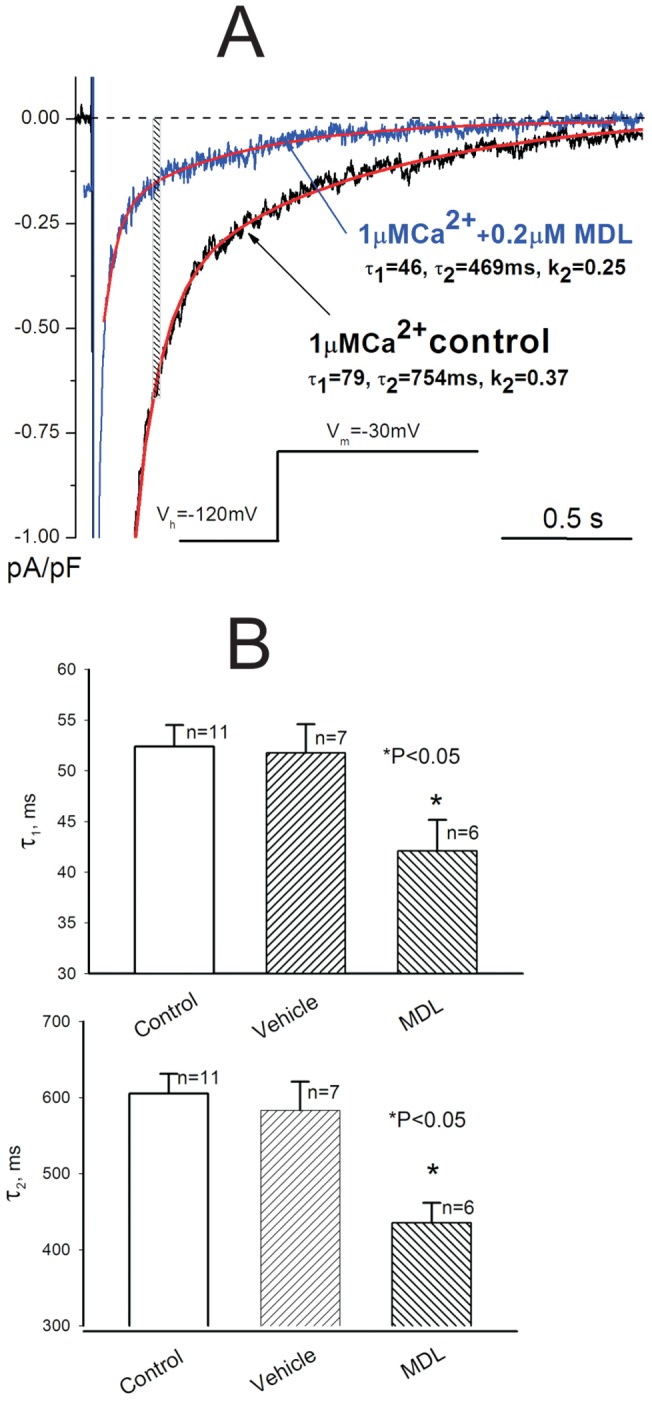
Calpain inhibitor MDL modulates I_NaL_ kinetics and amplitude in ventricular cardiomyocytes of ventricular myocytes of failing dog hearts. A. Representative raw traces were recorded at [Ca^2+^]_i_  = 1 μM in failing cardiac myocytes before (control) and after exposure to 0.2 μM of MDL. Exponential fits (Eq.1, see Methods) are shown by solid lines, together with their parameters. Inset shows voltage-clamp protocol. Vertical bar indicate the time window (200–220 ms of depolarization) used to evaluate I_NaL_ density. B. Statistical data for the decay time constants τ_1_ and τ_2_. Bars represent average data mean ±SEM, n – number of cells. Statistical significant difference *P<0.05 were evaluated by the Student's t-test.

Original I_NaL_ recordings were also analyzed to assess the current density (pA/pF), i.e. I_NaL_  =  (whole cell I_NaL_)/C_m_, where C_m_ is cell electric capacitance that was measured by a voltage ramp (16) in each cell. The I_NaL_ data points in the current-voltage relationships were measured as the averaged current density within 200–220 ms after depolarization onset (vertical bar in Fig. 1A). The steady-state activation (SSA) parameters were determined from the current-voltage relationships by fitting data points of the normalized current with the function [27]:
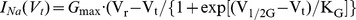
(2)Where G_max_ is a normalized maximum Na^+^ conductance, V_r_ is a reversal potential; V_½G_, and k_G_ are the midpoint and the slope of the respective Boltzmann function underlying the steady-state Na^+^ channel activation.

The steady-state inactivation (SSI) was evaluated by a double-pulse protocol with 2 s-duration pre-pulses (V_p_) ranging from –130 mV to −40 mV followed by a testing pulse to −30 mV. I_Na_ amplitudes were normalized to that measured at V_p_ =  −130 mV and the data points were fitted to a Boltzmann function A(V_p_):
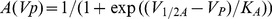
(3)


### 2.3. Numerical model simulation of calpain inhibition of effect in failing myocytes

We simulated effect of selective inhibition of calpain on AP shape and diastolic Ca^2+^ accumulation *in silico* using our previously reported modification of EC coupling model of failing canine ventricular myocyte (originally developed by Winslow et al. [28]). In short, our model has introduced a new detailed formulation of I_NaL_ lacking in the original model. This important model modification has allowed us to predict an important role of I_NaL_ to alter AP shape (increase AP plateau duration) and to contribute to diastolic Ca^2+^ accumulation in HF ventricular myocytes [17]. In short, in our in silico examinations we use I_NaL_ data measured under voltage clamp at 24°C and then apply Q_10_ factors to calculate model parameters for our full I_NaL_ description at 37°C. The details of the model parameters calculations have been described in our previous publications [17]. Specific parameter values of the present study are given in Table 1. The stair case phenomenon was simulated by assigning a relatively low [Ca^2+^] of 0.125 μM as an initial value in both network SR and junctional SR before application of stimulation pulse train (at 1 Hz or 1.5 Hz).

**Table 1:** Experimentally measured (Italic font) and derived (calculated) parameters of *I_NaL_* that was used in our numerical model simulations (at 37°C) of simultaneous dynamics of membrane potential and intracellular [Ca^2+^].

Para meter	Units	24°C		37°C	
		Control	MDL	Control	MDL
I_NaL,200ms_	pA/pF	*0.488*	*0.1661*	ND	ND
*τ_BM_*	Ms	*52.39*	*42.1*	18.7974	15.1054
*τ_LSM_*	Ms	*605.2*	*435.2*	217.145	156.149
*k_LSM_*	dimension less	*0.4088*	*0.49*	ND	ND
*k_BM_*	dimension less	*0.5912*	*0.51*	ND	ND
*I_max_*	pA/pF	2.43534	0.879164	4.12552	1.48932
*I_max_BM_*	pA/pF	1.81165	0.624718	3.06897	1.05828
*I_max_LSM_*	pA/pF	0.62369	0.254446	1.05655	0.431036
G*_NaL_max_*	mS/μF	ND	ND	0.03997	0.01505
*k_ A_*	mV	*5.2*	*5*	*5.2*	*5*
*V_ 1/2A_*	mV	*−74.86*	*−79.74*	*−74.86*	*−79.74*
*k_ G_*	mV	*6.4*	*6.9*	*6.4*	*6.9*
*V_ 1/2G_*	mV	*−42*	*−41*	*−42*	*−41*

ND: not defined. Parameter definitions were originally given in [17] and we also describe them in Section *3.4. ECC model predictions of physiological importance*.

### 2.4. Statistical Analysis

Multiple comparisons between treatment groups were made using one-way analysis of variance (ANOVA) followed by Bonferroni's post hoc test or by the non-pared Student's t-test if appropriate. Data are reported as mean±SEM. The significance of SSA or SSI changes were evaluated using F-test (StatMost, DataMost Corp., Salt Lake City, UT) for tabulated values predicted by the model (Eqs. 2, 3) at a confidence level of 0.95. Differences for both experimental data and model predictions were considered statistically significant for P<0.05.

### 2.5. Chemicals

Collagenas type II (291 U/mg) was from Worthington (Freehold, NJ). All other chemicals and enzymes, including calpain inhibitor MDL was purchased from Sigma (St. Louis, MO).

## Results

### 3.1. Calpain inhibitor MDL makes I_NaL_ decay faster in VCM from failing dog hearts

First we compare I_NaL_ decay in VCMs exposed to MDL with that in control, in the absence of the drug, at the intracellular Ca^2+^ = 1 μM (this intracellular Ca^2+^ was used in all experiments presented in this study). As it is shown in Fig. 1A in VCM exposed to MDL the I_NaL_ decay becomes faster than in control cell. Shown are raw traces along with the two-exponential fit (solid lines). Statistical data are given in Fig 1. B. Note that decay time course on the *I_NaL_* became significantly faster in MDL-treated cells as it is obvious from the reduction of the time constants τ_1_ (upper panel) and τ_2_ (lower panel).

### 3.2. In VCMs from failing dog hearts calpain inhibitor MDL decreases I_NaL_ density in wide range of the membrane potential without affecting the steady-state activation voltage-dependency

**Figure 2:**
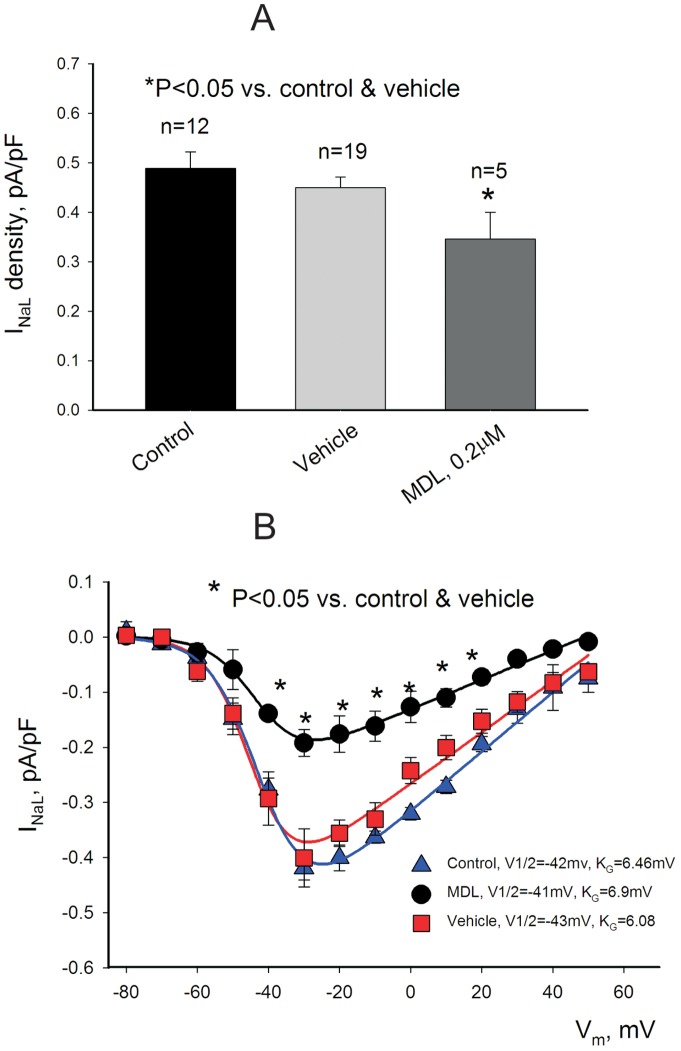
Calpain inhibitor MDL reduces I_NaL_ density without changes in the steady-state activation parameters in cardiomyocytes from dogs with heart failure. A. Statistical data for the I_NaL_ density in control (no vehicle), with the vehicle (DMSO), and in the presence of MDL (0.2 μM). The I_NaL_ density was measured at 200–220 ms after depolarization to –30 mV from the holding potential of –120 mV. B. Data points represent current-voltage relationship in control (blue triangles, red squares), and in the presence of MDL (black circles). The solid lines show theoretical curves of the steady-state activation (SSA, Eq.2, Methods) fitted to data point. MDL caused significant reduction of the maximum I_NaL_ conductance G_max_ from 5.5 (control) and 0.47(vehicle) to 0.27 pS/pF (MDL) (P<0.001, F-test). Other fit parameters (mid-point potential, V_1/2_, and the slope coefficient, k_G_, remained almost unchanged in augmented in these conditions (values are shown at the traces). In all these experiments depicted in the figure the intracellular [Ca^2+^]_i_ = 1 μM.

Treatment with MDL significantly reduced I_NaL_ density in VCMs compared with control and vehicle-treated cells (Fig. 2 A). The density was measured as an average current at the membrane potential of –30 mV within 200–220 ms after the depolarization onset (shown by the vertical bar in Fig. 1A). Fig. B shows an effect of MDL on I_NaL_ density in the wide range of the membrane potentials assessed in the IV relationship (dots). The solid lines represent theoretical fit to the Eq.2 (see Methods) with the aim to assess SSA parameters (Shown in the graph). The MDL does not affect the voltage-dependence of the SSA as it is evident of the mid-potential position and the slope of the curve, which we found to be unchanged. At the same time MDL reduced the maximum conductance, G_max_, for I_NaL_, which is expected because of the density reduction (See Fig. 2 Legend).

### 3.3. In VCMs from failing dog hearts calpain inhibitor MDL decreases does not affect the SSI of I_NaL_

**Figure 3:**
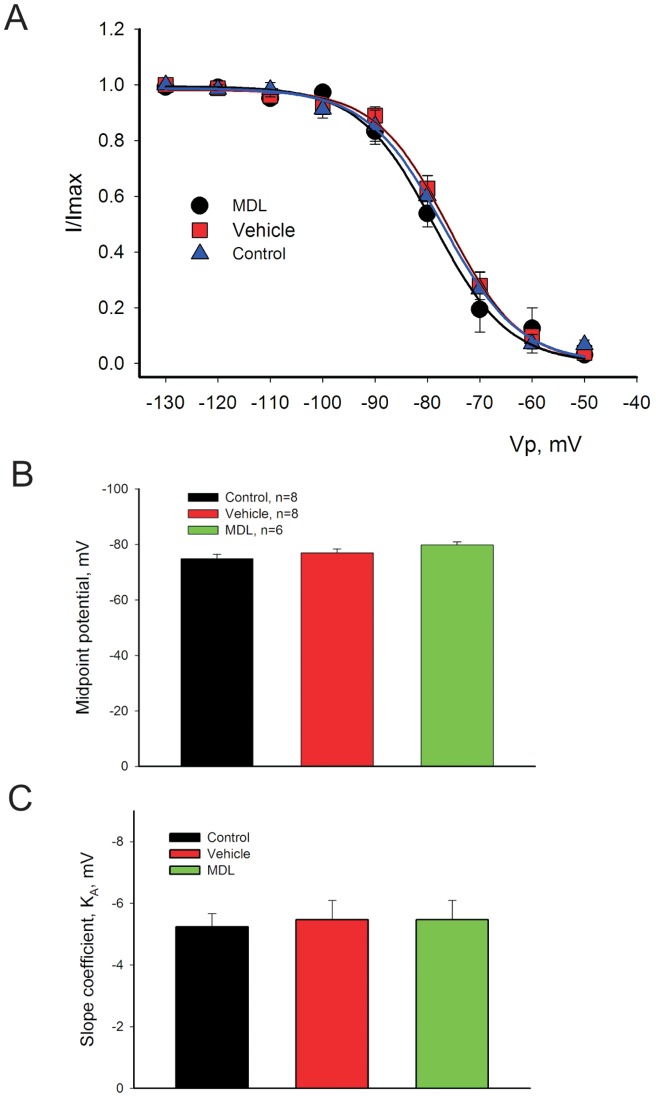
Calpain inhibitor MDL does not affect the steady-state inactivation (SSI) parameters in cardiomyocytes from dogs with the chronic heart failure. A. Data points and theoretical fit (solid lines, Eq. 3, Methods) for the steady-state inactivation in control (blue triangles, red squares), and in the presence of MDL(black circles). B, C- statistical analysis of SSI parameters; midpoint potential (V_1/2_), and slope (K_A_). There was no statistical difference between these groups. Data points were pooled from 8 cells. The intracellular [Ca^2+^] = 1 μM in these experiments.

Fig. 3A shows experimental points obtained by the two-pulse protocol along with the theoretical fit (solid lines, Eq.3 in Methods) for the SSI evaluation. There was no statistical difference (F-test) when the theoretical curves corresponding to a MDL, vehicle or control (no vehicle) were compared. Fig. 3B shows statistics for the SSI parameters, mid-point potential and slope coefficient K_A_. There was no significant difference for these parameters pointing to the absence of the effect on SSI by the MDL.

### 3.4. ECC model predictions of physiological importance

**Figure 4:**
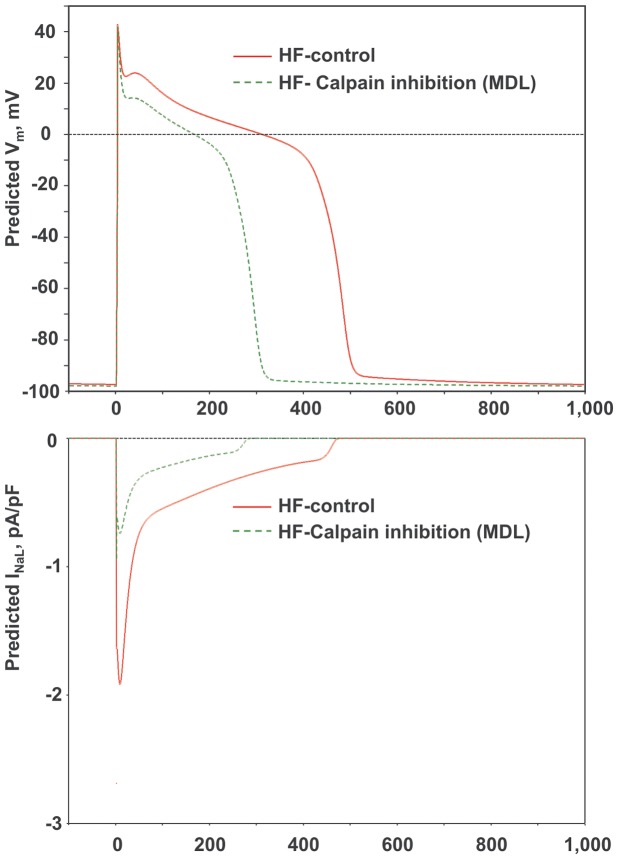
Numerical model simulations of action potentials (upper panel) and I_NaL_ (lower panel) of a ventricular myocyte from dog with HF in control and in response to calpain inhibition by MDL. All simulations are based on the respective changes of I_NaL_ parameters measured experimentally under voltage clamp (see Methods for the modeling details and Table 1). Pacing rate 1.0 Hz.

**Figure 5:**
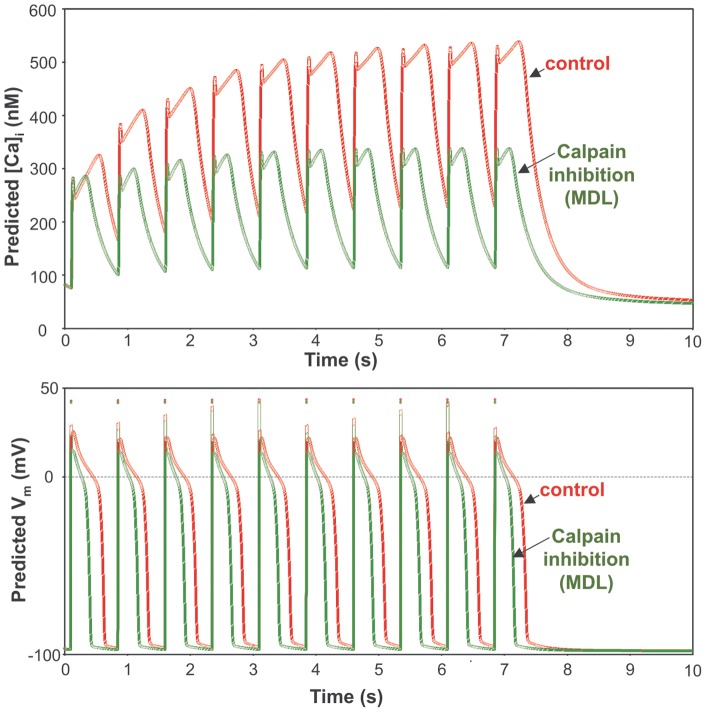
An in silico demonstration of physiological consequences of the I_NaL_ amplitude reduction and decay acceleration in the presence of calpain inhibition in ventricular myocytes of dogs with HF. Shown are model predictions for AP shape (lower panel) at 1 Hz pacing rate (steady-state) and cytosolic [Ca^2+^] (upper panel) in a train of 10 consecutive excitation pulses applied with a rate of 1.5 Hz. Note a substantial diastolic [Ca^2+^]_i_ accumulation at the end of the pulse train in control but not in the presence of MDL. At the same time AP duration significantly decreased by MDL during the pulse train (lower panel).

We tested if these experimentally measured I_NaL_ changes caused by the calpain inhibitor are physiologically significant in HF myocytes. Using I_NaL_ characteristics measured in the present study, we carefully calculated all I_NaL_ parameters (Table 1) required for numerical modeling of I_NaL_ effects as we previously established for VCMs of dog failing heart [17]. Specifically, I_NaL,200ms_ is the density of I_NaL_ measured at 200 ms after membarne depolarization onset; *τ_BM_* and *τ_LSM_* are *τ_1_* and *τ_2_*, respectively in Equation 1; *k_BM_* and *k_LSM_* are *k_1_* and *k_2_*, respectively in Equation 1; parameters *k_ G_, V_ 1/2G_,* and *k_ A_, V_1/2A_* are defined in Equations 2 and 3, respectively. We corrected I_NaL_ decay constants and maximum amplitude density for 37°C using Q_10_ factors (2.2 and 1.5, respectively) as we described in our previous studies [29], [30] as follows: *τ_37_  =  τ_24_* ⋅2.2^(24–37)/10^ and *I_max_37_  =  I_max_24_* ⋅1.5^(37–24)/10^. Finally, *I_max_  =  I_max_BM_ + I_max_LSM_* and *G_NaL_max_* are the maximal total I_NaL_ peak current density and conductance, respectively. Fig. 4 upper panel shows results of the in silico test of how MDL-induced I_NaL_ changes affect AP shape and duration at a physiological temperature of 37°C at a pacing rate of 1 Hz. Note that simulated APs are shorter with lower plateau in VCMs treated by MDL. Lower panel of Fig. 4 shows the prediction of I_NaL_ dynamics (profile) during the AP in control and after MDL treatment. Our simulations show that the amplitude and duration of I_NaL_ become substantially smaller in MDL-treated cells vs. control (untreated) cells. Fig. 5 upper panel shows in silico simulations of the intracellular [Ca^2+^] dynamics in VCMs of failing heart. In response to the pulse train stimulation with the rate of 1.5 Hz, the diastolic Ca^2+^ level gradually increases in control conditions. The MDL-induced changes in I_NaL_ amplitude and decay kinetics almost completely eliminate this diastolic Ca^2+^ accumulation pattern. Lower panel of Fig. 5 shows simultaneous AP simulations for this condition. Note shorter AP with the lower plateau similarly to that shown in Fig. 4.

## Discussion

For the first time we demonstrate at the single cell level that I_NaL_ alterations in amplitude and decay kinetics associated with chronic HF can be rescued by calpain inhibition. Our in silico simulations also demonstrate that the calpain modulation of I_NaL_ is physiologically important in HF myocytes, specifically, calpain inhibition greatly improves the myocyte function by reducing the action potential duration, and intracellular diastolic Ca^2+^ accumulation in the pulse train.

The calpain family is a group of cysteine proteases unique in their dependency on calcium to attain functionally active forms [31]. Calpain is involved in a wide range of Ca^2+^-regulated cellular processes such as signal trasduction, secretion, cell proliferation, differentiation and apoptosis [11]. Calpain deregulation resulting from the impaired Ca^2+^ handling is one of the important mechanisms for the pathological processes such as apoptosis and necrosis, reperfusion-induced heart stunning, ischemia and hypoxia, hyperthrophy and heart failure, and atrial fibrillation [11], [32]. Therefore calpain inhibition is considered a therapeutic strategy targeting multiple disease states [32].

Our findings thus suggest a novel cellular and molecular mechanism to modulate NaCh that could be targeted to prevent pathophysiological consequences related to the augmented I_NaL_ in HF. There are some indications of the involvement of calpain into ion channel gating regulation, namely L-type Ca^2+^ channels [33], [34]. In this context the calpain inhibition may serve to improve Ca^2+^ handling in failing heart and may be considered as a novel approach to modulate I_NaL_ current its related arrhythmias, and improve contractility [1], [2]. Below we discuss possible cellular and molecular mechanisms of calpain effect on I_NaL_.

### 4.1. Calpain and fodrin cytoskeleton

Fodrin-based cytoskeleton, an important element of the NaCh microenvironment in heart, is a dynamic structure that is altered under a variety of pathological conditions (e.g. ischemia or heart failure [13], [14], [35]). The role of the fodrin-based cytoskeleton in I_NaL_ modulation has been confirmed in our previous studies [1]. It has also been shown that fodrin breakdown that occurs in some disease states featuring poor Ca^2+^ handling can be mediated by calpain [15], [35]. Therefore prevention of Ca^2+^- induced fodrin cytoskeleton degradation will likely improve Ca^2+^ handling in HF.

### 4.2. Interplay between Ca^2+^, CAM/CaMKII cascade and calpain

It has been shown that I_NaL_ depends on the [Ca^2+^]_i_ signaling cascade in the way that increased [Ca^2+^]_i_ binds to EF-hand motif on NaCh C-terminal domain [6], [36], [37] or via activating CaM/CaMKII cascade resulting in the augmented and slowed I_NaL_ [6], [7]. This is very important mechanism of I_NaL_ regulation in HF because Ca^2+^ homeostasis is impaired in this disease stage. Inhibition of calpain results in reduced density of I_NaL_ despite of the presence of high [Ca^2+^]_i_ that works in the opposite direction [6], [7]. At the same time SSI and SSA parameters remain unchanged pointing to the fact that all channels are available for I_NaL_ and that the parameters of SSI and SSA depend on [Ca^2+^]_i_, rather than on calpain-dependent proteins. Indeed we have shown that the direct binding of Ca^2+^ to NaCh [37] (likely to E–F hand domain of NaCh C-terminus) is responsible for shifts of the half membrane potential of SSI voltage dependence towards depolarizing potentials [6]. Therefore, reduction of I_NaL_ density produced by MDL likely results from reduced probability of NaCh transitions into different modes (burst and late scattered modes) that are involved in I_NaL_ formation [29]. The faster I_NaL_ decay in the presence of calpain inhibition (Fig. 1) also indicates that gating of these modes is also affected by calpain.

### 4.3. Interplay between calpain and NaCh β-subunits

It has been shown that besides the main pore-forming α subunit of NaCh [37], the β_2_-subunit of NaCh is attached to the subsarcolemmal cytoskeleton [38]. Therefore prevention of cleavage of fodrin by calpain may stabilize the cytoskeleton and enhance the β_2_-subunit dependent modulation of I_NaL_ that we have recently reported [10]. We have shown that reduction of β_2_ expression by the siRNA increased I_NaL_ density and delayed its decay in VCMs from dogs with HF, i.e. very similar to that caused by the increased [Ca^2+^]_i_ [6] (via activation of calpain) and opposite to that caused by the calpain inhibition by MDL shown herein.

### 4.4. Physiological relevance of Ca^2+^-calpain signaling to modulate I_NaL_ and to improve contractility and rhythm of failing heart

It has been established that I_NaL_ plays an important role in both electrical and contractile (via Ca^2+^ handling) deficiencies caused by chronic HF [1], [2]. Then an important question is whether the magnitude of the effect of calpain inhibition on I_NaL_ reported in the present study is physiologically relevant. To address this question, we have carefully measured and analyzed specific characteristics of I_NaL_ in control and in the presence of MDL (Table 1) and then integrated them into our recently published ECC numerical model for ventricular cardiomyocytes of the failing dog [10], [17]. As it is evident from Figs. 4 and 5, MDL substantially reduces effects of I_NaL_ on AP duration, which is known to increase in HF [1]. The resultant decrease of I_NaL_ during AP plateau is observed as I_NaL_ becomes scaled (decreased) by about a factor of two during most of the plateau. This substantial “scaling” contributes not only to in AP duration shortening but also in to that AP plateau becomes substantially lower. This insight does not directly follow from the voltage clamp data and even not from AP simulations, because of a complex interplay of many Na^+^- and Ca^2+^- dependent mechanisms in ventricular cells reproduced by the dynamic ECC model.

Since shorter AP plateau and a smaller inward current during AP plateau are associated with less incidence of EADs [20], [39], one expected beneficial effect of calpain inhibition is also to reduce the probability of the EADs [17], a major mechanism for the triggered arrhytmia. Recently we have demonstrated that the augmented and slowed I_NaL_ in HF contributes to the diastolic [Ca^2+^]_i_ accumulation in VCM of failing hearts during the pulse train [17]. Reduction of I_NaL_ by the MDL significantly reduces this accumulation [Ca^2+^]_i_ as it is predicted in silico (Fig. 5). Previously it has been demonstrated that delayed afterpotentials are linked to the diastolic Ca^2+^ accumulation associated with I_NaL_, [17], [40]. Therefore this predicted effect of MDL to prevent the diastolic [Ca^2+^]_i_ accumulation indicates, in turn, that calpain inhibition can reduce probability of occurrence of the DADs.

### 4.5. Conclusion

Based on our present results with the specific calpain inhibitor MDL in ventricular cardiomyocytes isolated from failing dog hearts, we conclude that Ca^2+^-dependent calpain activation is able to strongly modulate I_NaL_ density and kinetics in failing myocardium. We illustrate in silico that the range of this modulation is physiologically relevant and remarkable as the calpain inhibition substantially improves (shortens) AP duration and prevents diastolic Ca^2+^ accumulation. Therefore, this Ca^2+^-dependent signaling cascade may serve as a plausible target to regulate I_NaL_ and its related electrical and contractile deficiencies in failing heart.
